# The role of TREM2 in sepsis. Reply.

**DOI:** 10.1172/JCI189219

**Published:** 2025-02-17

**Authors:** Siqi Ming, Xingyu Li, Jingxian Shu, Xi Huang, Yongjian Wu

**Affiliations:** 1 Department of Laboratory Medicine, Guangdong Provincial Hospital of Chinese Medicine, Zhuhai Hospital, Zhuhai, Guangdong Province, China.; 2 Center for Infection and Immunity, the Fifth Affiliated Hospital, Sun Yat-sen University, Zhuhai, Guangdong Province, China.

**Keywords:** Inflammation, Innate immunity

**The authors reply (1):** We are thankful for the comments from Zhu et al. concerning our recent paper by Ming et al. (2). Included below is a response to their questions.

With regard to the survival rate and cytokines in the cecal ligation and puncture (CLP) mouse model, it has been reported that the variability of the CLP technique, including the length of ligated cecum and needle size, can induce a range of severity, thereby permitting the induction of both acute and chronic sepsis (3). We established acute lethal CLP models in wild-type (WT) and TREM2 systematic–knockout (TREM2^–/–^) mice using an 18-gauge needle and 3/4 ligation to observe the survival rate, and almost all WT mice were dead within 72 hours. Meanwhile, for the survival rate assay, more than 10 mice were employed per group (see Supporting Data Values file in ref. 2). However, Zhang et al. conducted survival rate assays in WT and TREM2^–/–^ mice by establishing a chronic sublethal CLP model with a 22-gauge needle and 1/3 ligation and more than 85% of WT mice survived until 72 hours (4). It is known that the underlying inflammatory response and the outcome (survival rate) vary with the severity grade of CLP-induced sepsis (3). A publication in 2009 has demonstrated that therapeutic interventions that show highly protective effects in mid-grade sepsis are ineffective in a more severe form of sepsis (5). In our study, we observed reduced mortality in TREM2^–/–^ mice during a severe CLP polymicrobial sepsis model, bacterial sepsis model, and LPS-induced acute endotoxemia model. Consistently, improved survival rates in TREM2^–/–^ mice were also observed by Gawish et al. (6), similar to our results from an LPS acute endotoxemia model, and mice used in their study were also provided by Prof. Marco Colonna (Washington University, St. Louis, Missouri, USA). The differences in the effect of TREM2 knockout on mortality between two severe CLP models are reasonable, because the varieties of pathogenesis and involved immune cells in different-severity sepsis may lead to the complexity of TREM2 function. Meanwhile, the differences in gut-derived microbiota from different-severity CLP may also influence the outcome of disease mediated by TREM2. We reveal that in severe CLP and bacterial sepsis models or LPS-induced acute endotoxemia, TREM2 displays a detrimental role and promotes inflammation, while in mid-grade sepsis, TREM2 may play different roles, which needs further investigation. In addition, although cytokine expression was also evaluated in the work of Zhang et al., they primarily examined the levels of inflammatory cytokines in heart tissue, while we tested inflammatory cytokine levels in serum, liver, and lung tissue. Notably, tissue-specific effects of TREM2 on immune cell function and inflammation have been reported and also in part explain the discrepancy between the two studies.

As for the organ injuries and infiltration of inflammatory cells, in the work by Zhang et al., they established a CLP model and detected the infiltration of immune cells in heart tissue after 3 days and 7 days (*n* = 4 WT mice and *n* = 5 TREM2^–/–^ mice). In our study, we evaluated lung infiltration of inflammatory cells in WT and TREM2^–/–^ mice (*n* = 5 per group). Although no significant difference in neutrophil number was observed between WT and TREM2^–/–^ mice, they found a reduced proportion of cardiac macrophages in TREM2^–/–^ mice (Extended Data Figure 5F in ref. 4), which is consistent with our observations in lung tissue (Figure 2C in ref. 2). In addition, the authors seem to focus on the cardiac injury indicators in mice (*n* = 5–11 per group) and injuries of other organs were not assessed in their study, while we evaluated the injuries of lung, liver, and kidney (*n* = 5–10 per group). Notably, data in our study were verified from at least three independent experiments. Moreover, in our study, nonlethal CLP was employed for the detection of inflammatory cell infiltration at 72 hours, as we described in the Methods.

## Version 1 02/17/2025

Electronic publication
